# Sequential versus concurrent chemotherapy for adjuvant breast cancer: does dose intensity matter?

**DOI:** 10.1038/bjc.2017.176

**Published:** 2017-06-22

**Authors:** N LeVasseur, S K Chia

**Affiliations:** 1 Department of Medical Oncology, British Columbia Cancer Agency, Vancouver, Canada

While the role of adjuvant chemotherapy for patients with early-stage breast cancer has been clearly established (Early Breast Cancer Trialists’ Collaborative Group (EBCTCG), 2005), a number of important clinical questions remain unanswered in certain groups. Although it has been shown that long-term outcomes are improved with anthracycline and taxane-containing regimens relative to anthracyclines alone (Early Breast Cancer Trialists’ Collaborative Group (EBCTCG), 2012), no single regimen has been consistently found to be superior, resulting in an area of significant clinical equipoise. Newer strategies for treatment dosing and schedules have also been explored to optimise the delivery of effective drugs in an attempt to improve clinical outcomes. However, most large adjuvant trials are comprised of node-positive patients particularly as it relates to the question at hand (Shao *et al*, 2012), leaving some uncertainty regarding the magnitude of benefit in high-risk node-negative patients.

In this issue of the *British Journal of Cancer*, Mavroudis *et al*, (2017), report the interim results of the phase III Hellenic Oncology Research Group (HORG) trial comparing sequential *vs* concurrent administration of an anthracycline and a taxane in a population of high-risk node-negative breast cancer patients. Of 658 women, 329 (50%) were randomly assigned to receive epirubicin 90 mg m^−2^ for four cycles followed by docetaxel 75 mg m^−2^ for four cycles (sequential) or epirubicin 75 mg m^−2^ plus docetaxel 75 mg m^−2^ for six cycles (concurrent). Definition of high-risk node-negative for trial purposes was either T2 size or greater, or T1 size, and at least two of the following criteria: ER/PgR negative, grade 3, Ki67>40% or lymphovascular/perineural invasion. The study population included 44% being premenopausal, 64% hormone-receptor-positive patients, 56% with a tumour size of greater than 2 cm (T2) and 53% with a grade 3 histology.

The HORG investigators are to be congratulated for conducting a well-designed trial accruing over 12 years in 10 centers with exceptional follow-up data. The focus of the study specifically in a ‘high-risk’ node-negative breast population is a particular uniqueness and strength of this randomised controlled study. Planned dose intensity in both arms were optimal, as treatment was delivered on time in over 96% of cycles, and dose reduction was required in less than 5% of delivered cycles (primary prophylaxis with growth factors were mandatory in the concurrent arm and optional in the sequential arm). In an interim analysis, with 70.5 months of median follow-up, the trial suggests a non-significant difference in disease-free survival (DFS) favouring the use of sequential anthracyclines and taxanes over the concurrent regimen (92.6% *vs* 88.2%HR 1.591, 95% CI 0.990–2.556; *P*=0.055). This difference appeared to be primarily driven by the hormone-receptor-negative cohort for DFS (92.2% *vs* 83.6% HR 2.214, 95% CI 1.068–4.593; *P*=0.033) and for OS (HR 3.369; 95% CI 0.94–12.081, *P*=0.062). Though this was a *post hoc* analysis, in multivariate analyses there was a significant interaction between treatment arms and hormone receptor status.

This trial of sequential *vs* concurrent administration of an anthracycline and a taxane regimen adds to the foundation of evidence regarding the optimal chemotherapy strategy for high-risk node-negative breast cancer patients. The first observation from this study is the relatively overall favourable prognosis for this patient population. A 5-year DFS between 88–92% is dramatically better than the initial statistical assumption of the study of 65%. This lower event rate is the reason why this planned interim analysis should be considered to be the final analysis. With one-third of the study population having hormone-receptor-negative disease, these results will likely remain favourable over time. Even more optimistic is that in the predominantly triple-negative cohort (as HER-2 was unknown in this study), the 5-year DFS in the sequential arm was 92.2%. This is evidence to the fact that not all treated triple-negative breast cancers demonstrate poor outcomes.

What perhaps is the most interesting aspect of the study results is to decipher why the sequential arm appeared to out-perform the combination arm. This raises the question of the relative importance of cumulative dose *vs* dose intensity for adjuvant chemotherapy in early-stage breast cancer. The combination arm in fact delivered higher cumulative doses of both epirubicin (450 mg m^−2^) and docetaxel (450 mg m^−2^) compared to the sequential arm (epirubicin 360 mg m^−2^ and docetaxel 300 mg m^−2^). Thus, perhaps once the threshold of total dose is surpassed, higher cumulative doses do not add to efficacy but rather do add to cumulative toxicity (such as cardiac toxicity and neuropathy for anthracyclines and taxanes, respectively). Calculating the dose intensity of each agent (measured as mg m^−2^ per week) provides some insight into the biological rationale for the differential activity. The sequential arm received 20% relatively higher dose intensity (30 mg m^−2^ per week) of the anthracycline compared to the combination arm (25 mg m^−2^ per week). There was no difference in dose intensity for docetaxel across the arms (25 mg m^−2^ per week). This concept validates the finding from the NEAT trial (National Epirubicin Adjuvant Trial) performed in 2021 patients across 65 UK centres that a higher dose intensity confers greater favourable long-term outcomes (Earl *et al*, 2008).

Two further concepts that this study could build on to further improve outcomes for adjuvant chemotherapy in early-stage HER-2-negative breast cancer are dose density and the genomic selection of tumours for greater sensitivity to chemotherapy. Higher dose intensity can be achieved by either delivering a greater dose per cycle or by reducing the intervals between cycles (known as dose density). The principle behind dose density relates to the Norton-Simon hypothesis and the Gompertzian model that proposes an increase in growth kinetics following each cycle of cytoreductive chemotherapy (that is, smaller tumours grow faster than larger tumours)—thus a rationale for reducing the time between cycles (Simon and Norton, 2006). The concept was tested in two landmark trials (CALGB 9741 and GONO-MIG-1), in which the same drugs (anthracycline+/−taxane) and same doses were given per cycle but at different cycle frequencies (Citron *et al*, 2003; Venturini *et al*, 2005). The CALGB 9741 study results supported dose density and the GONO-MIG-1 trended so, as interesting however was the greater benefit seen in the ER negative cohorts (Del Mastro *et al*, 2005)–much like the HORG trial.

Two-thirds of the trial population on the HORG trial had tumours that were hormone-receptor-positive (ER+). It is now well recognised that not all ER+ tumours benefit from adjuvant chemotherapy, and that genomic assays can help to differentiate cohorts that derive greater *vs* negligible benefit from adjuvant chemotherapy (Paik *et al*, 2006). It is conceivable that an enrichment in the population of the HORG trial by a genomic assay selection could have led to a greater differential activity for the sequential arm of the study. It is also acknowledged, however, that the currently available assays have not yet demonstrated an ability to differentiate greater benefit between different chemotherapy regimens based on the assay results or categorisation of results (Mamounas *et al*, 2012).

In conclusion, the trial reported by Mavroudis *et al.* in this issue of the *British Journal of Cancer* adds further to the foundation of evidence that supports the recommendation of a sequential, appropriately dose-intensified anthracycline-taxane-based regimen in early-stage breast cancer. It is unlikely that further dramatic gains will be made in future by slight modifications of cytotoxic chemotherapeutic regimens or schedules. Rather the focus of future clinical trials should be investigating the use of genomic assays to enrich patient populations enroled into adjuvant systemic trials, and the addition of targeted agents (either in combination or in sequence) to standard-of-care therapy to further improve clinical outcomes in early-stage breast cancer.

## References

[bib1] Citron ML, Berry DA, Cirrincione C, Hudis C, Winer EP, Gradishar WJ, Davidson NE, Martino S, Livingston R, Ingle JN, Perez EA, Carpenter J, Hurd D, Holland JF, Smith BL, Sartor CI, Leung EH, Abrams J, Schilsky RL, Muss HB, Norton L (2003) Randomized trial of dose dense versus conventionally scheduled and sequential versus concurrent combination chemotherapy as post-operative adjuvant treatment of node positive primary breast cancer: first report of Intergroup Trial C9741/Cancer and Leukemia Group B Trial 9741. J Clin Oncol 21: 1431–1439. 12668651 10.1200/JCO.2003.09.081

[bib2] Del Mastro L, Bruzzi P, Nicolo G, Cavazzini G, Contu A, D’Amico M, Lavarello A, Testore F, Castagneto B, Aitini E, Perdelli L, Bighin C, Rosso R, Venturini M (2005) HER-2 expression and efficacy of dose dense anthracycline containing adjuvant chemotherapy in breast cancer patients. Br J Cancer 93: 7–14. 15970926 10.1038/sj.bjc.6602660 PMC2361477

[bib3] Earl HM, Hiller L, Dunn JA, Bathers S, Harvey P, Stanley A, Grieve RJ, Agrawal RK, Fernando IN, Brunt AM, McAdam K, O’Reilly S, Rea DW, Spooner D, Poole CJ (2008) NEAT: National Epirubicin Adjuvant Trial-toxicity, delivered dose intensity and quality of life. Br J Cancer 21: 1226–1231. 10.1038/sj.bjc.6604674 PMC2570521 18797468

[bib4] Early Breast Cancer Trialists' Collaborative Group (EBCTCG) (2005) Effects of chemotherapy and hormonal therapy for early breast cancer on recurrence and 15-year survival: an overview of the randomised trials. Lancet 365: 1687–1717. 15894097 10.1016/S0140-6736(05)66544-0

[bib5] Early Breast Cancer Trialists' Collaborative Group (EBCTCG) (2012) Comparisons between different polychemotherapy regimens for early breast cancer: meta-analyses of long-term outcome among 100,000 women in 123 randomised trials. Lancet 379: 432–444. 22152853 10.1016/S0140-6736(11)61625-5 PMC3273723

[bib6] Mamounas EP, Tang G, Paik S, Baehner FL, Liu Q, Jeong JH, Kim SR, Butler SM, Jamshidian F, Cherbavaz DB, Sing AP, Shak S, Julian TB, Lembersky BC, Wickerham DL, Costantino JP, Wolmark N (2012) Association between the 21-gene Recurrence Score® results and benefit from adjuvant paclitaxel in node positive, ER positive breast cancer patients: Results from NSABP B-28. In: Cancer Res 72(24 Supplement): S1-10–S1-10.

[bib7] Mavroudis D, Saloustros E, Boukovinas I, Papakotoulas P, Kakolyris S, Ziras N, Christophylakis C, Kentepozidis N, Fountzilas G, Rigas G, Varhalitis I, Kalbbakis K, Agelaki S, Hatzidaki D, Georgoulias V (2017) Sequential vs concurrent epirubicin and docetaxel as adjuvant chemotherapy for high-risk, node-negative, early breast cancer: an interim analysis of a randomised phase III study from the Hellenic Oncology Research Group. Br J Cancer (e-pub ahead of print 22 June 2017; doi:10.1038/bjc.2017.158. 10.1038/bjc.2017.158 PMC5584474 28641315

[bib8] Paik S, Tang G, Shak S, Kim C, Baker J, Kim W, Cronin M, Baehner FL, Watson D, Bryant J, Costantino JP, Geyer CE Jr, Wickerham DL, Wolmark N (2006) Gene expression and benefit of chemotherapy in women with node-negative, estrogen receptor-positive breast cancer. J Clin Oncol 24: 3726–3734. 16720680 10.1200/JCO.2005.04.7985

[bib9] Shao N, Wang S, Yao C, Xu X, Zhang Y, Lin Y (2012) Sequential versus concurrent anthracyclines and taxanes as adjuvant chemotherapy of early breast cancer: a meta-analysis of phase III randomized control trials. Breast 2: 389–393. 10.1016/j.breast.2012.03.011 22542064

[bib10] Simon R, Norton L (2006) The Norton-Simon hypothesis: designing more effective and less toxic chemotherapeutic regimens. Nat Clin Pract Oncol 3: 406–407. 16894366 10.1038/ncponc0560

[bib11] Venturini M, Del Mastro L, Aitini E, Baldini E, Caroti C, Contu A, Testore F, Brema F, Pronzato P, Cavazzini G, Sertoli MR, Canavese G, Rosso R, Bruzzi P (2005) Dose dense adjuvant chemotherapy in early breast cancer patients: results from a randomized trial. J Natl Cancer Inst 97: 1724–1733. 16333028 10.1093/jnci/dji398

